# Supplementary material for: Modelling porcine NAFLD by deletion of leptin and defining the role of AMPK in hepatic fibrosis
Source: Cell Biosci. 2023 Sep 13;13:169. doi: 10.1186/s13578-023-01124-1 (PMC10498639; doi:10.1186/s13578-023-01124-1)
Supplement: Supplementary file 1 — Additional file 1. Additional figures and tables. [file 13578_2023_1124_MOESM1_ESM.pdf]

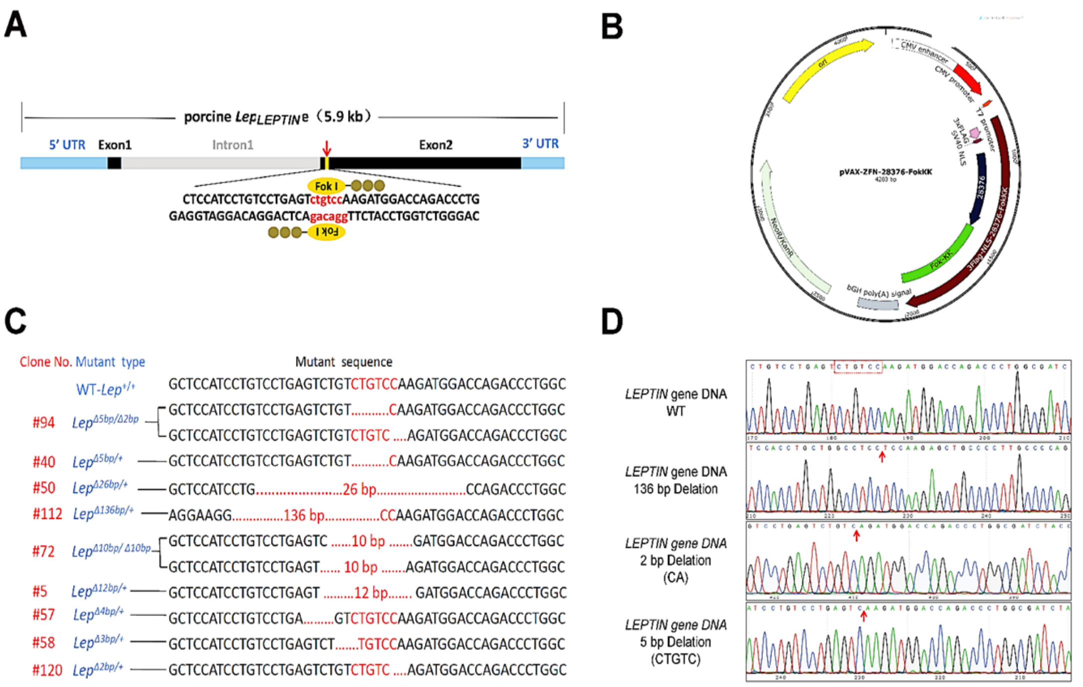

Supplemental Figure 1. Generation of *LEPTIN* mutant pigs.

A. Porcine *LEPTIN* gene targeting scheme (targeting site in red). **B.** Construction of ZFN target vector. **C.** Positive clones and observed *LEPTIN* mutations. **D.** Sequence analysis of *LEPTIN* mutants. Peak maps shown mutated base pairs in *LEPTIN* gene on three different chromatids.

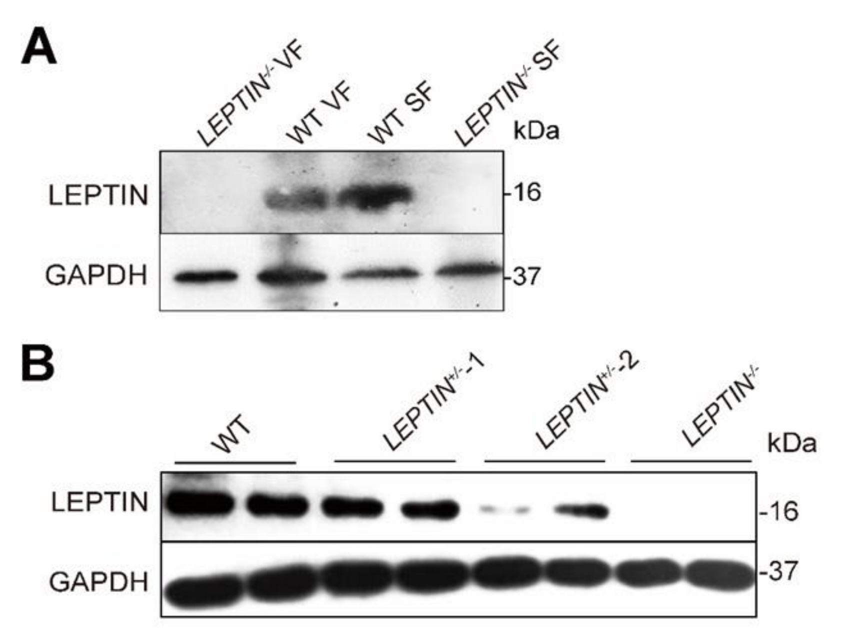

Supplemental Figure 2. Detection of Leptin in mutant pigs.

A. The level of Leptin in subcutaneous fat and visceral fat was measured by WB. **B.** WB of Leptin expression in serum.

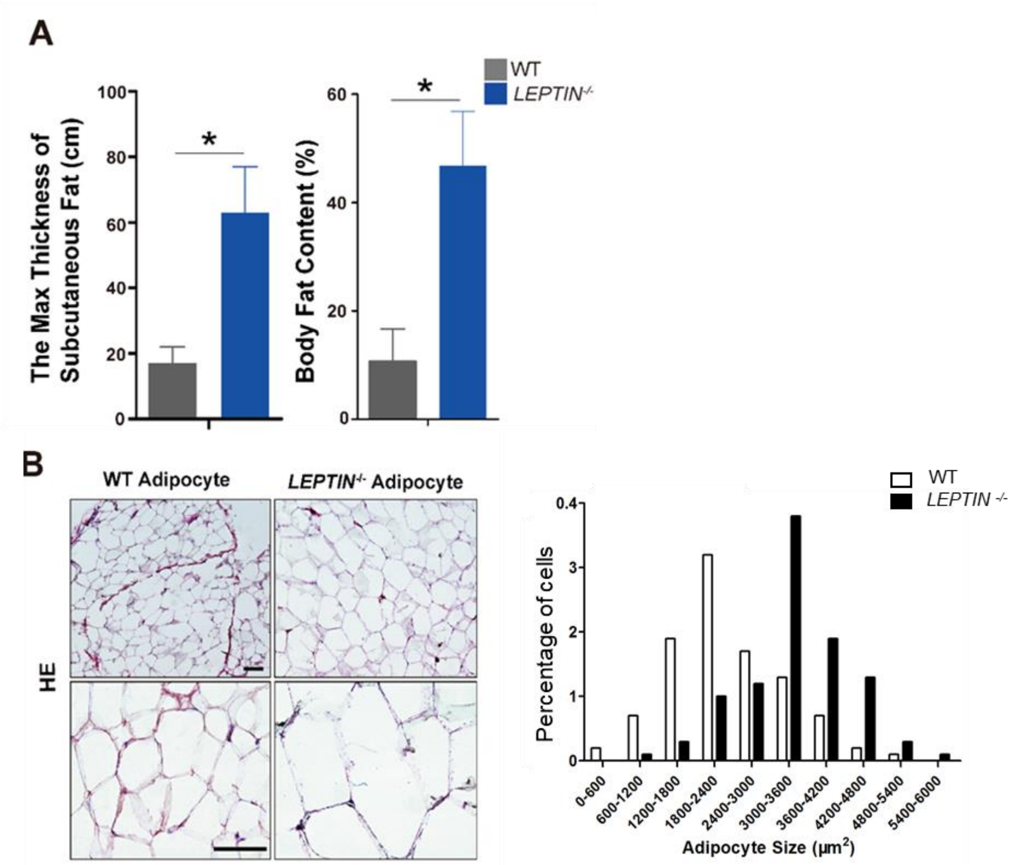

Supplemental Figure 3. Analysis of obesity phenotypes in LEPTIN-/- pigs.

A. Maximum thickness of subcutaneous fat and the percentage of body fat in *LEPTIN*$^{-/-}$ and WT pigs. **B.** HE staining of adipose tissue and the analysis of adipocyte size. Bar=100μm. The bars represent the mean ± SD; NS, non-significant. *P<0.05. Bar=20μm.

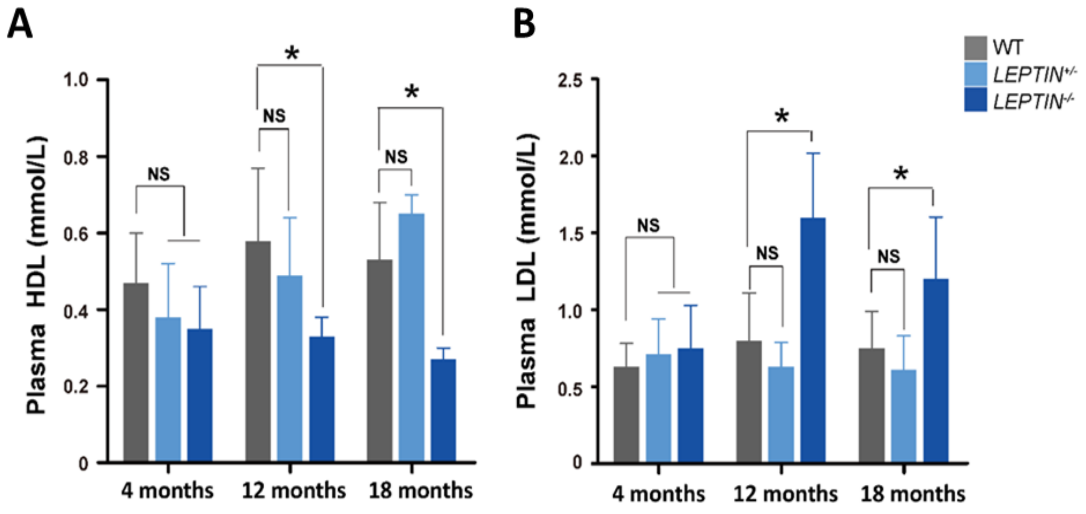

Supplemental Figure 4. Analysis of Serum HDL and LDL in LEPTIN-/- pigs.
Serum high density lipoprotein (HDL) **(A)** and low density lipoprotein (LDL) concentrations **(B)**. The bars represent the mean ± SD; NS, non-significant. *P<0.05. Bar=20μm.

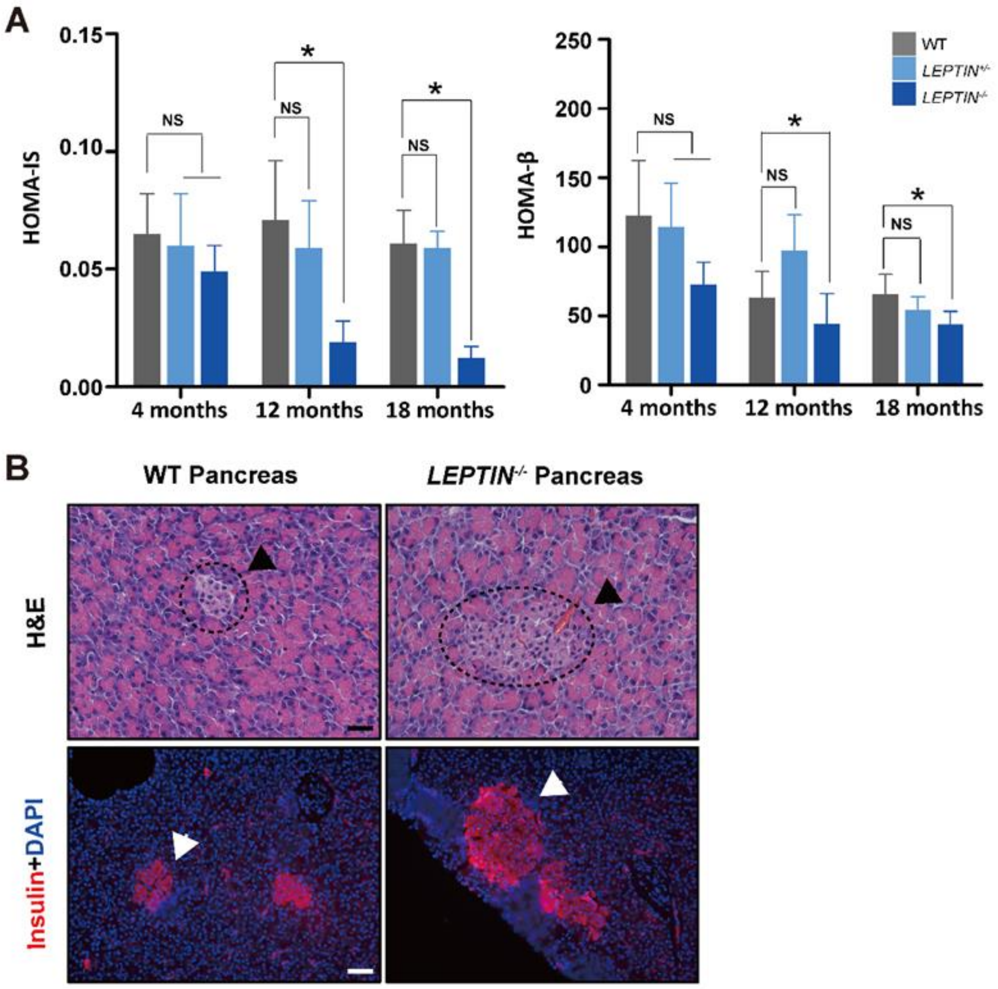

Supplemental Figure 5. Analysis of the type II diabetes in LEPTIN-/- pigs.

A. HOMA-IS analysis to evaluate insulin sensitivity and HOMA-β analysis to evaluate function of β cells for pigs at different ages. **B.** H&E staining and insulin immunofluorescence of pig pancreatic tissue. Dotted box and arrows indicate islet and β cells (insulin positive). The bars represent the mean ± SD; NS, non-significant. *$P<0.05$. Bar=20μm.

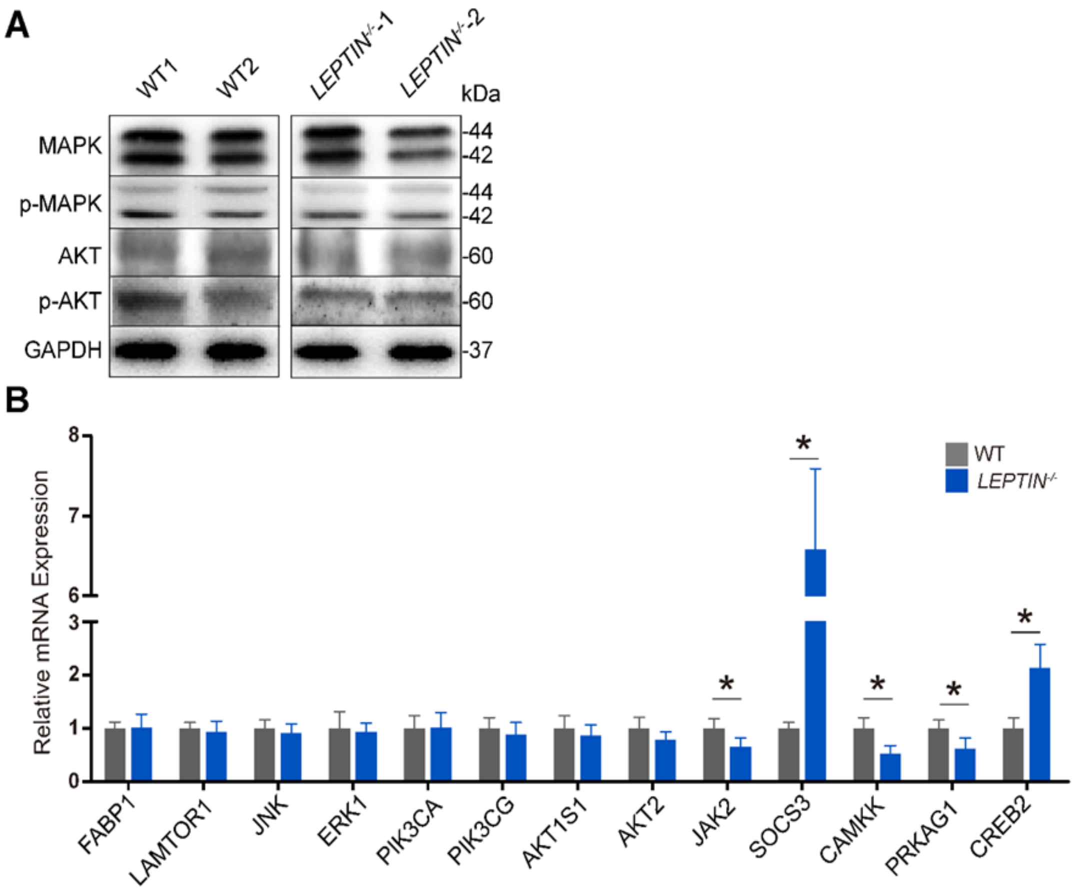

Supplemental Figure 6. The effects of LEPTIN deficiency on mTOR, MAPK and PI3K-AKT pathways in pig livers.

A. WB analysis of mTOR, PI3K-AKT, MAPK pathway related proteins. **B.** Expression of mTOR, MAPK, and PI3K-AKT pathway related genes detected by qPCR. The bars represent the mean ± SD; *$P<0.05$.

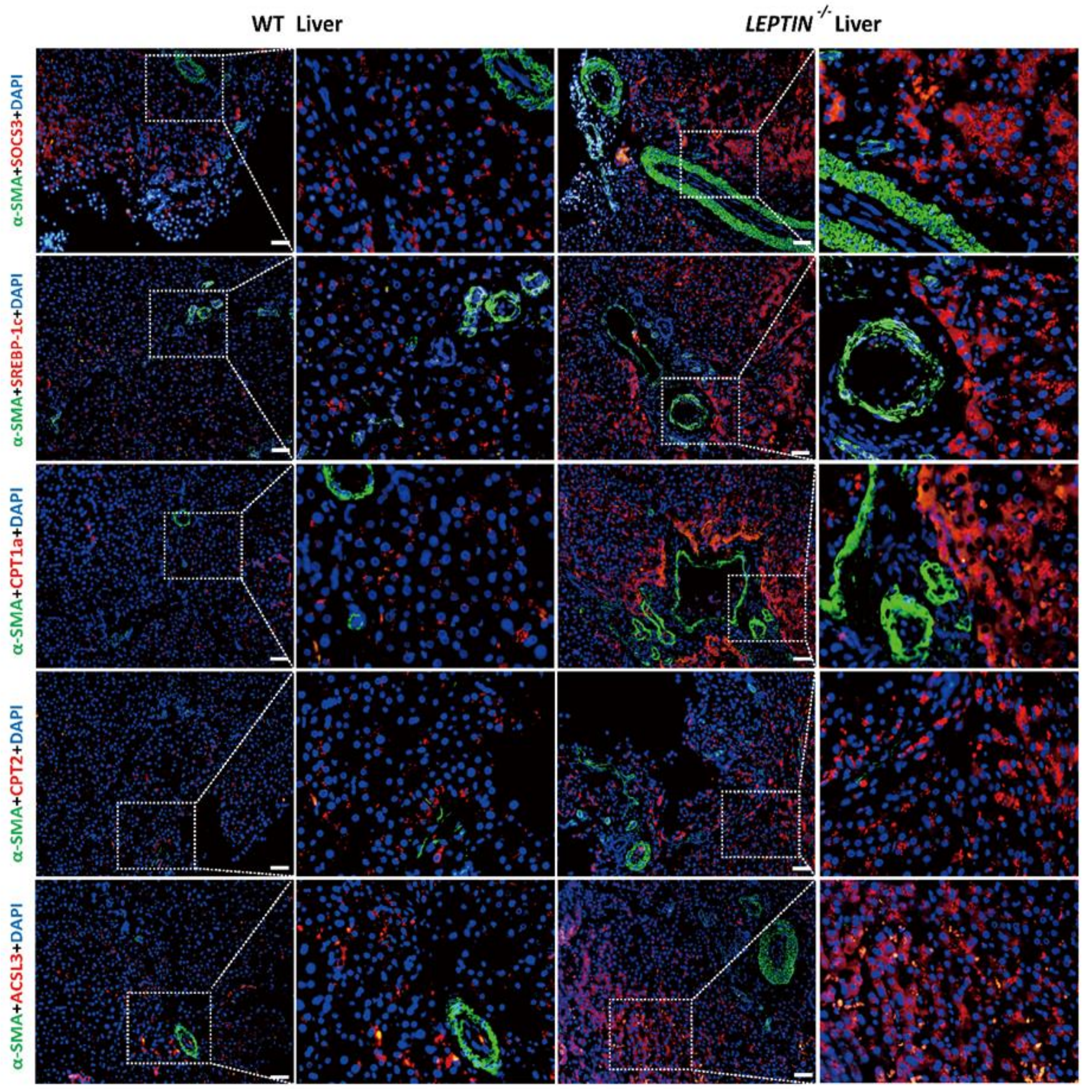

Supplemental Figure 7. Histological detection of proteins affected by JAK-STAT signaling.

Immunofluorescence staining of JAK-STAT pathway related proteins involved in FFA synthesis and β-oxidation process. The white dashed box indicated the enlarged area. Bar=100μm.

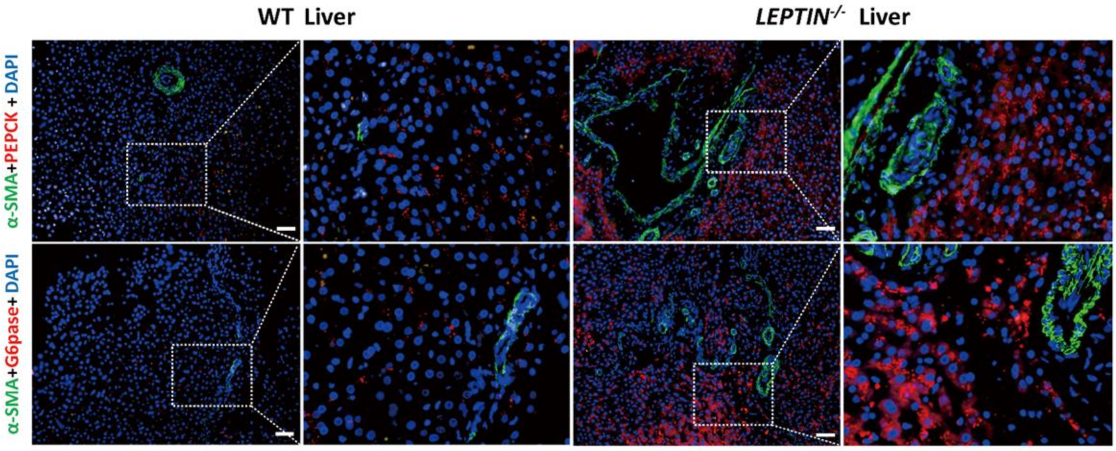

Supplemental Figure 8. Histological detection of proteins affected by AMPK signaling.

Immunofluorescence staining of AMPK pathway related proteins involved in gluconeogenesis. The white dashed box indicated the enlarged area. Bar=100μm.

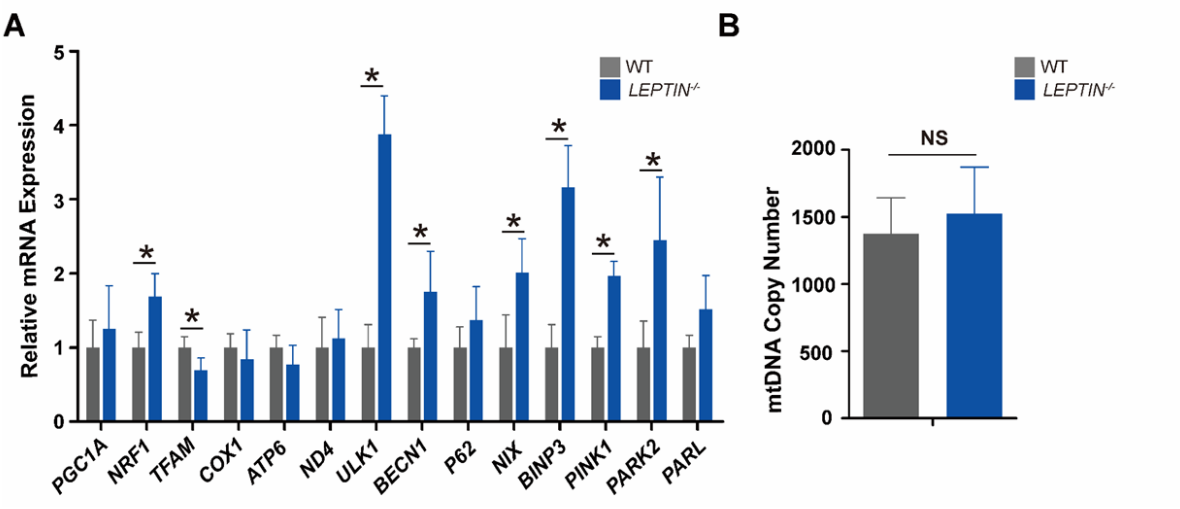

Supplemental Figure 9. Effects of *LEPTIN* knockout on mitochondrial function in pig livers.

A. Expression analysis of mitochondrial synthesis and autophagy related genes by qPCR. **B.** Measurement of Mitochondrial DNA copy numbers. The bars represent the mean ± SD; NS, non-significant. *P<0.05.

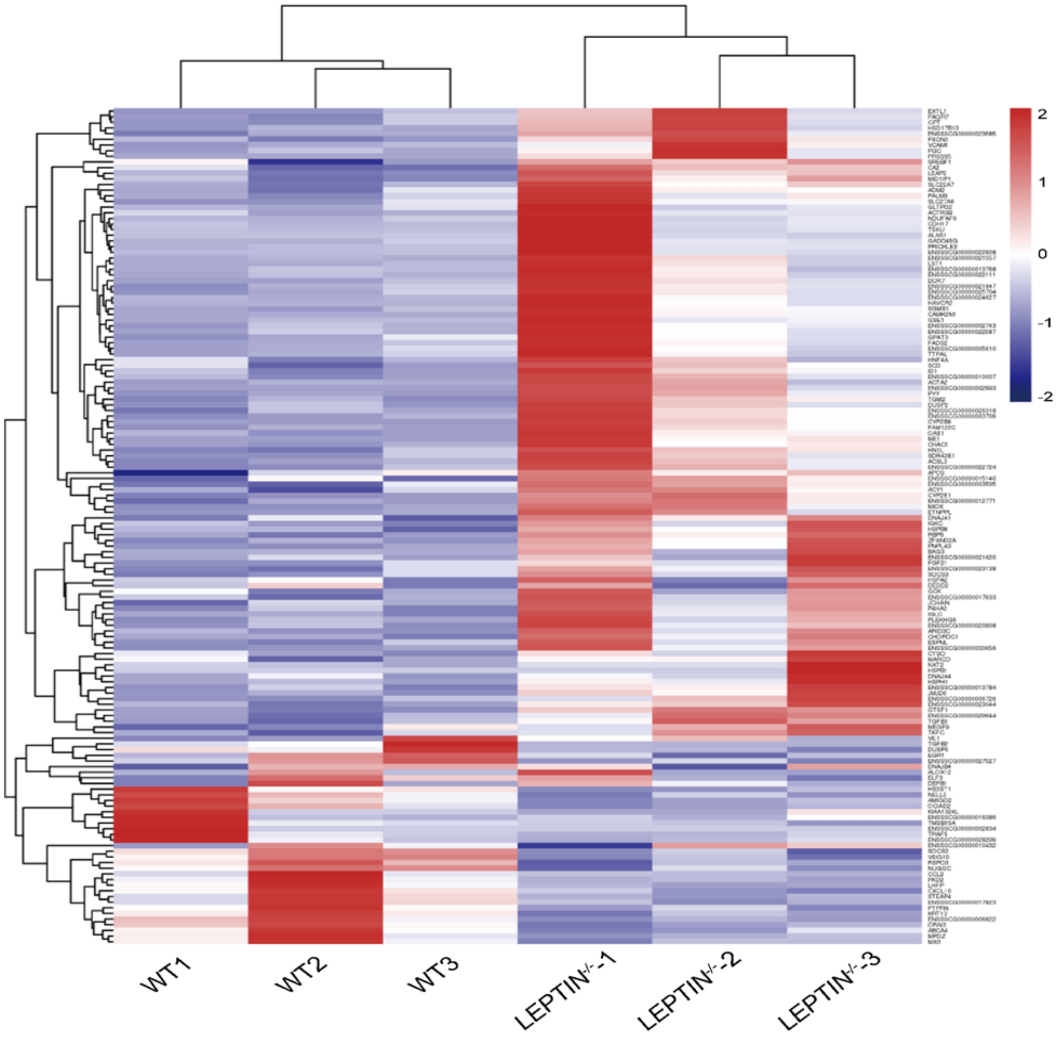

Supplemental Figure 10. Heatmap of DEGs between LEPTIN-/- and WT pig livers. Heatmap displaying the relative expression of DEGs in liver samples from three WT and *LEPTIN*$^{-/-}$ pigs assessed via RNA-seq. Red indicates up-regulated and blue indicates down-regulated genes.

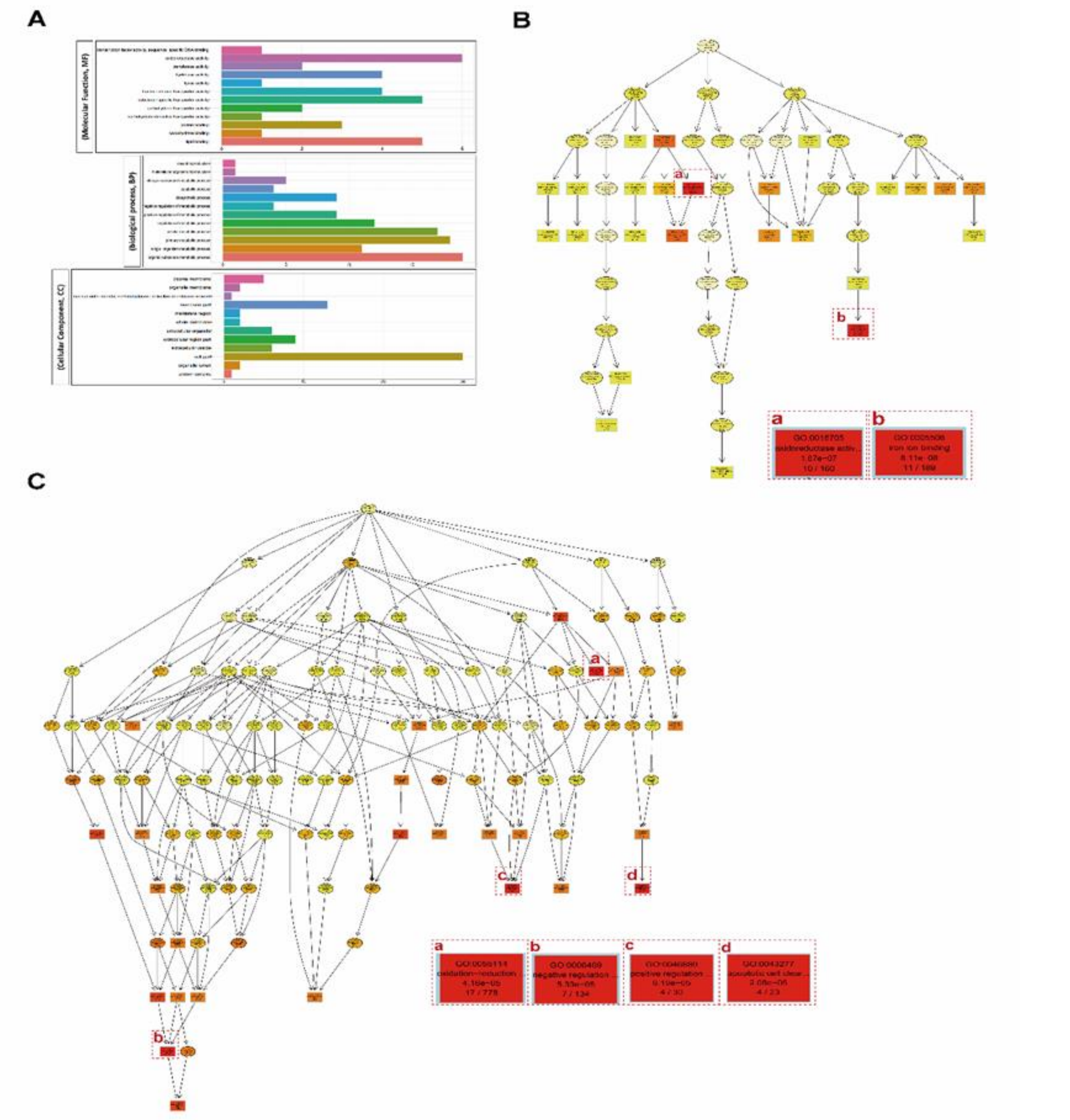

Supplemental Figure 11. GO terms enriched for DEGs.

A. Enrichment of molecular function, biological process and cell component GO terms. The ordinate represents the entry names for different functions, processes and components, and the abscissa represents the number of genes enriched on that entry. **B.** Molecular functional pattern of enriched GO terms. Each node represents a GO term, and the darkness of the color indicates the degree of enrichment. The term name and corrected *P*-value are shown for each node. The node terms **(a)** and **(b)** represent the two most highly enriched of the molecular functions. **C.** Biological process pattern of enriched GO terms. The node terms **a**, **b**, **c** and **d** are the four most highly enriched biological processes.

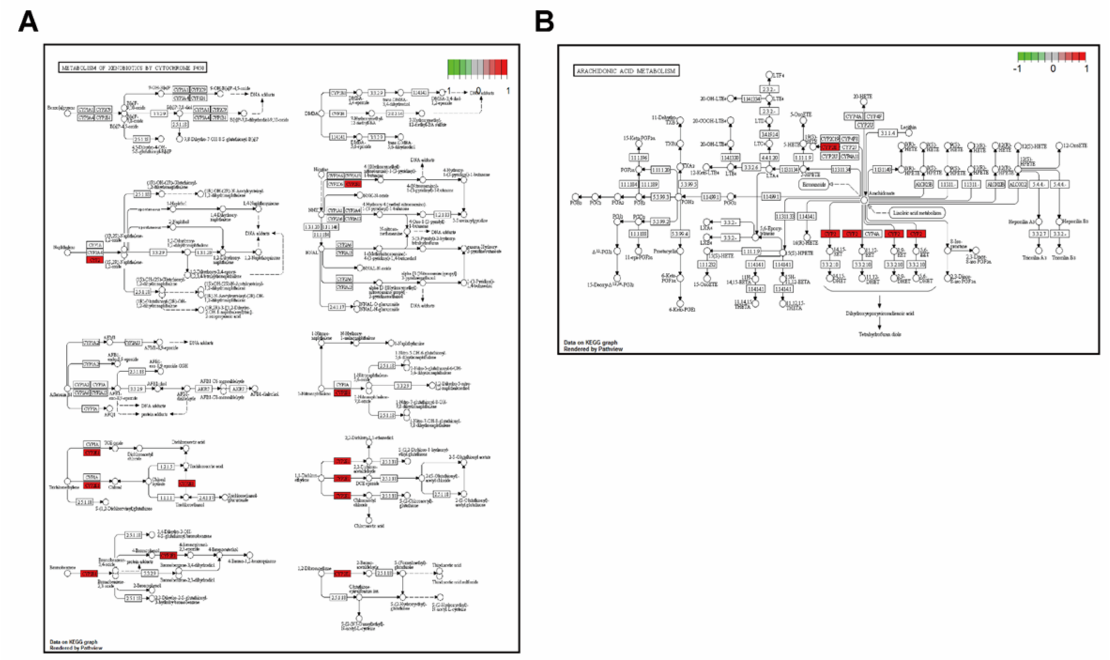

Supplemental Figure 12. P450 enzyme (CYP2) related KEGG pathways enrichment for DEGs.

Boxes represent molecular compounds, solid arrows represent chemical reactions, and dotted arrows represent indirect reactions. Red boxes represent DEGs. **A.** Metabolism of xenobiotics by cytochrome P450 (00980). **B.** Arachidonic acid metabolism pathway (00590).

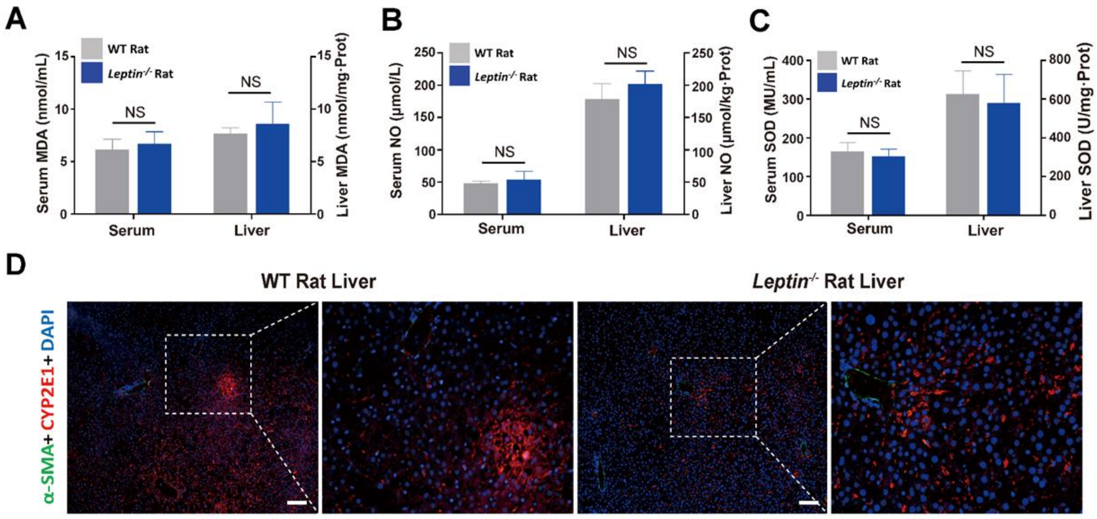

Supplemental Figure 13. Oxidative stress analysis in Leptin-/- rat liver.
A-C. Analysis of oxidative stress markers, MDA **(A)**, NO **(B)** and SOD **(C)**, in the Leptin-/- rat liver and serum. n=3. The bars represent the mean±SD; NS, non-significant. **D.** Immunofluorescence staining of CYP2E1. The white dashed box indicated the enlarged area. Bar=100μm.

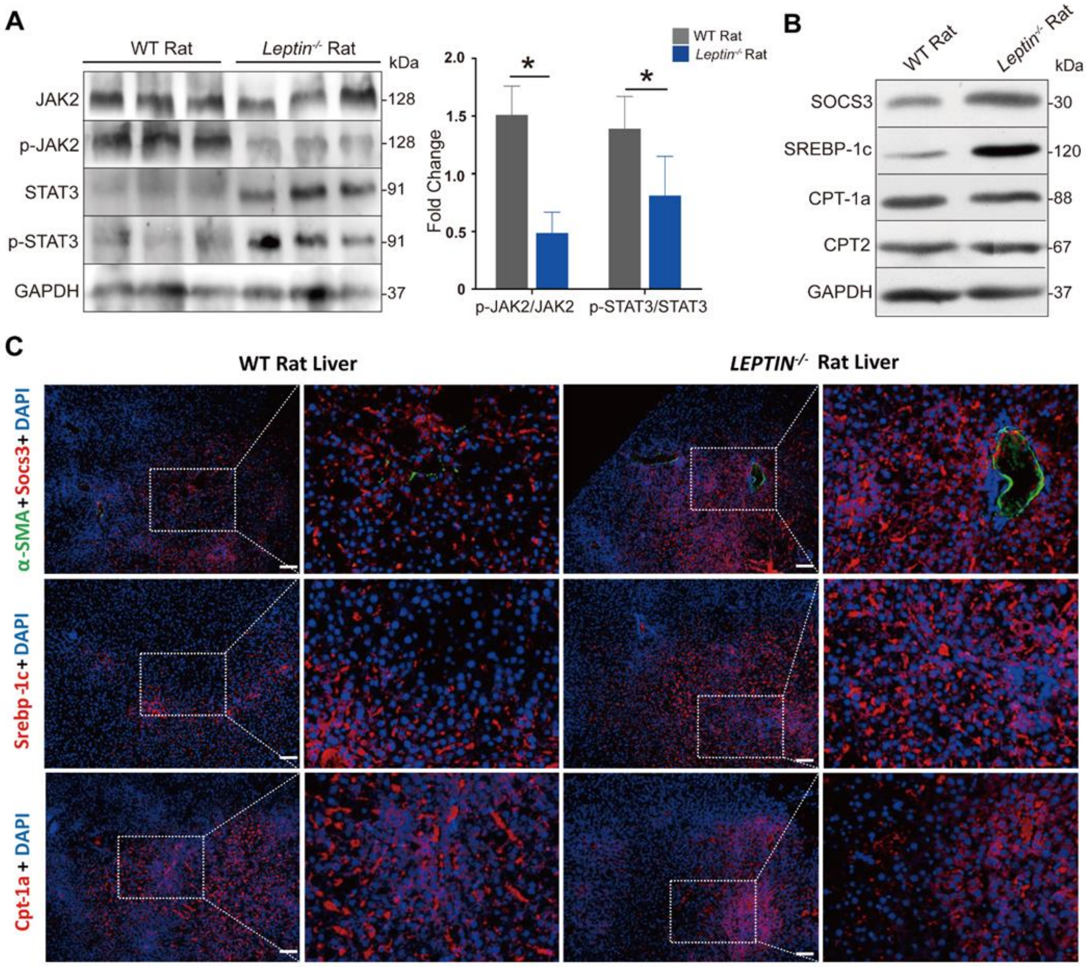

Supplemental Figure 14. Analysis JAK-STAT pathway related signaling and histological detection of proteins affected by JAK-STAT pathway in Leptin-/- rats.

A. WB analysis of CYP2E1 in rat livers. **B.** WB analysis of JAK-STAT pathway related proteins in rat livers. Gray scale quantitative analysis of JAK2/p-JAK2 and STAT3/p-STAT3 protein expression. The bars represent the mean ± SD; *P<0.05. NS, non-significant. **C.** Immunofluorescence staining of JAK-STAT pathway related proteins involved in FFA synthesis and β-oxidation processes. The white dashed box indicated the enlarged area. Bar=100μm.

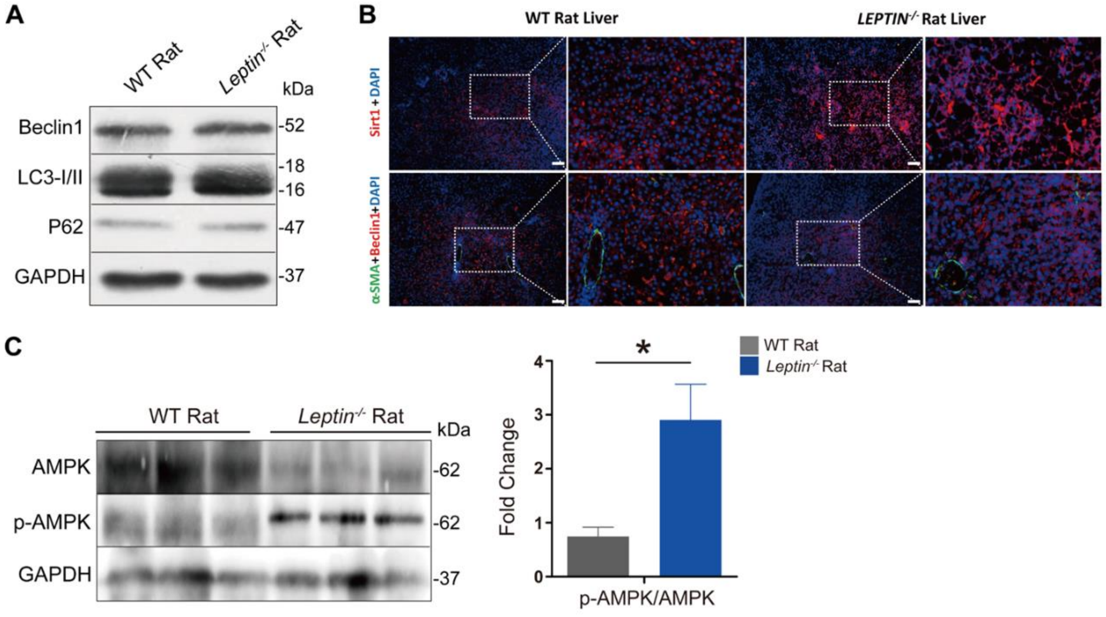

Supplemental Figure 15. Analysis AMPK pathway related signaling and histological detection of proteins affected by AMPK pathway in Leptin-/- rats. **A.** WB analysis of mitochondrial autophagy related markers. **B.** Immunofluorescence staining of AMPK pathway related proteins involved in mitochondrial autophagy. The white dashed box indicated the enlarged area. Bar=100μm. **C.** WB analysis of AMPK and p-AMPK proteins. Gray scale quantitative analysis of AMPK/p-AMPK protein expression. n=3/group. The bars represent the mean±SD; *P<0.05. NS, non-significant.

Sequence location	Alignment length	Mis-match	Gapopens	Identity (%)	Mutation
**chr18:21204359-21204400**	42	0	0	100.0	**Yes**
**chr3:143300508-143300548**	44	4	4	79.55	**No**
**chr2:53593108-53593148**	41	4	3	82.93	**No**
**chr14:54951351-54951389**	40	3	3	77.5	**No**
**chr3:88219157-88219193**	40	8	1	72.5	**No**
**chr4:106294931-106294969**	39	6	1	74.36	**No**
**chr17:51681679-51681714**	36	8	1	75	**No**
**chr2:75293422-75293457**	36	8	0	77.78	**No**
**chr9:132547427-132547462**	36	7	1	77.78	**No**
**chr2:8133431-8133461**	35	2	3	80	**No**
**chr15:34396511-34396545**	35	6	1	74.29	**No**
**chr1:226121719-226121752**	34	6	1	76.47	**No**
**chr13:745664-745697**	34	7	0	79.41	**No**
**chr3:37458176-37458208**	34	5	2	76.47	**No**
**chr3:126567384-126567416**	34	4	2	76.41	**No**
**chr9:148747377-148747410**	34	5	1	82.35	**No**

Supplemental Table 1. Analysis of off-target mutations in *LEPTIN*$^{-/-}$ pigs.

Sequence locations were obtained by whole pig genome BLAST with ZFN cut site sequences. The yellow part represents the target site section, with each homologous section and the chromosome position on the genome listed below. “No” meant that there was no genetic mutation detected.

KEGG ID	P value	Term
04930	0.00121462728629849	Type II diabetes mellitus
01040	0.00220721598093938	Biosynthesis of unsaturated fatty acids
04910	0.0046728761188535	Insulin signaling pathway
03320	0.00676667349145339	PPAR signaling pathway
00980	0.022849447585731	Metabolism of xenobiotics by cytochrome P450
00061	0.026029982698255	Fatty acid biosynthesis
00524	0.026029982698255	Butirosin and neomycin biosynthesis
00982	0.0304824410474875	Drug metabolism - cytochrome P450
05144	0.0332236017422863	Malaria
00590	0.0375102219892134	Arachidonic acid metabolism

Supplemental Table 2. KEGG pathways were enriched for DEGs.

The analysis of pathway enrichment was based on the KEGG pathway analysis ($P<0.05$), and hypergeometric tests were used to screen the pathways with significant enrichment.

Protein Name	Item No.	Manufacturer
ACSL3	sc166374	Santa Cruz Biotechnology, US
ACSL5	sc365478	Santa Cruz Biotechnology, US
AMPK	sc74461	Santa Cruz Biotechnology, US
Beclin1	sc11427	Santa Cruz Biotechnology, US
CPT-1a	ab128568	Abcam, UK
CPT2	sc20671	Santa Cruz Biotechnology, US
CYP2E1	ab28146	Abcam, UK
GAPDH	2118S	Cell Signaling Technology, , US
G6Pase	sc398155	Santa Cruz Biotechnology, US
HNF-4α	sc374229	Santa Cruz Biotechnology, US
JAK2	sc278	Santa Cruz Biotechnology, US
LC3- Ⅰ/Ⅱ	AL221	Beyotime Biotechnology, CN
LEPTIN	ab16227	Abcam, UK
LEPTIN-Rb	sc8325	Santa Cruz Biotechnology, US
P62	ab101266	Abcam, UK
p-AMPK	2535S	Cell Signaling Technology, US
Parkin	sc74461	Santa Cruz Biotechnology, US
PEPCK	sc166778	Santa Cruz Biotechnology, US
PINK1	sc33796	Santa Cruz Biotechnology, US
p-JAK2	sc16566	Santa Cruz Biotechnology, US
p-Parkin	sc135704	Santa Cruz Biotechnology, US
p-STAT3	sc8001R	Santa Cruz Biotechnology, US
SIRT1	sc15404	Santa Cruz Biotechnology, US
SOCS3	ab16030	Abcam, UK
SREBP-1c	ab3259	Abcam, UK
STAT3	sc8019	Santa Cruz Biotechnology, US
α-SMA	ab5694	Abcam, UK

Supplemental Table 3. The list of antibodies used in this study.

In this table, the item No. and manufacturer of all antibodies used in this study for WB and immunofluorescence staining are listed in details.

Gene	Forward primer (5'-3')	Reverse primer (5'-3')
FABP1	CCAAGGTCGTCCAGAATGAG	CTGAACCACTGTCTTGACCTT
FASN	GTCCTGCTGAAGCCTAACTC	TCCTTGGAACCGTCTGTG
ACC1	GGCCATCAAGGACTTCAACC	ACGATGTAAGCGCCGAACTT
ELOVL6	CCTGGTTTCTGCTCTGTATGCT	CAGCACTAATGGCTTCCTCAGTT
SCD1	CCCAGCCGTCAAAGAGAA	CGATGGCGTAACGAAGAAA
PPARA	TTGTCGTTTCCACAAGTGCC	TTTTCAGACCTTGGCATGCG
TNFA	AACCTCAGATAAGCCCGT	AGGACCTGGGAGTAGATGA
Il6	ACCAGGAACGAAAGAGAG	CAGTAGCCATCACCAGAA
NFKB	GCATCCAGACCAACAACAAC	ATGGGATGAG AAAGGACAGG
Il1B	AACGTGCAGTCTATGGAGT	GAACACCACTTCTCTCTTCA
Mcp1	CCGAAGCTTGAATCCTCATC	TAGCAGCAGGTGACTGGAGA
TGFB	GTGGCTGTCCTTTGATGT	CGTGGAGTGTGTTATCTTTG
ACTA2	CGGGATCCAAACAGGAATACGA CGAAG	CGCAAGCTTCAGGAATGATTTGGA AAGGA
LRAT	TGATGCCCGACATCCTATTG	ATGTCGGCTC CGTAGGCGAA G
CTHRC1	TGGGTGGAACTCAGTGTCTCGAATCA	CCTTCAGTCAAAACTGGTTTCAA
COL1A1	CAGAACGGCCTCAGGTACCA	CAGATCACGTCATCGCACAAC
TIMP1	AGCCAGGAGTTTCTCATAGC	TCACAGCCAGCAGCATAG
SERPINE1	TTGCCCTTGTGTGCTTGTTAG	AAAGAGAGGAGCAATGGGGTT
CTGF	GTGTGACGAGCCCAAGGA	GGGCCAAACGTGTCTTCCA
ACAT1	GCTGATGCTGCTGTAGAT	ACCATATTCTCCTTGCTTCA
ACAT2	ATACCAAGGAGCGAATCC	CCTCTTCTGCTTGTCCCAAC
ACADM	AGGAGCCATTGATGTGTGC	CTGCTTTGGTCTTTATACCAGCTA
ACADS	GGAGGCTCAGGTGAAGAAG	GTGTAGGCCAGGTAATCCAG
ACADL	TGTCTCCAGCTGCATGAAACGA	AGCTGCACACAGTCATAAGCCA
ACSL1	TCGCAGTGGCATCATTAG	GACCATCAGCCTCACTTTT
ACSL3	GGATCCACAGGACTTCCAAA	CGGCATCCATGAGAAAGAC
ACSL5	CCAAGGAGATATTCAGTTGCT	GGCTTCACTTTGTACCTTATCAT
ACOT7	TCGCCCATGTGCATCGGCG	TTCTCGGACATGACGTGGACC
ACOT8	GTGGCTGCCTATATCTCGG	CGTGGAACCA CATGGAGTGG
ACOT12	GAGGAAGGAACGGTTTCCAC	AGGCGGCTTGCAGAAATAGTA
CPT1A	GCATTTGTCCCATCTTTCGT	GCACTGGTCCTTCTGGGATA
CPT1B	ACTGTCTGGGCAAACCAAAC	CTTCTTGATGAGGCCTTTGC

CPT1C	ATGGGAATGCGGCCCTTATG	AGGTGGTGGATGTGGTCTCTG
CPT2	CAAGGCCTACCCTCTGGATA	GCTCACAATCTTCCCGTCTT
G6PD	AAGCCAAGCGAAGGTGTGAGC	AAGCATTCAGCCAACA
FBP1	TCCACCGCACGCTGGTCTAT	CCAGTCCTCCTGCCTTCTCCAT
PEPCK1	TCAGCACGACTCCAGCCTTCA	GCTCAAGCAGTCTGGGCATTCT
PEPCK2	ACAGGAGGTTCGTGACATTCGG	GTGGTGCTGTGCTCACTTGCTA
HNF4A	ATCGCCACCATCGTCAA	CCTCACCCTTTCCACTACCA
SIRT1	TTGATCTTCTCATTGTTATTGGGTC	ACTTGGAATTAGTGCTACTGGTCTTA
CYP2A19	GGAGAAGAAGAATCCTGACACCG	GCCTCCACATCCGGTTTCTT
CYP2B22	GCTGTGGTGGAGCCAATCTT	CCGAAGGGTCTTCCAACGTT
CYP2E1	ACCCTGAGATACGGGCTCCTAA	ACGGCATCCAGGTAGGGCAT
CYP2C49	TCCCCAACCCAGAGGTGTT	CCTTCTCCCACACACCTTCGTT
FABP1	CCAAGGTCGTCCAGAATGAG	CTGAACCACTGTCTTGACCTT
LAMTOR1	TTCCGCTCGCACAGACGAG	CTCCATGCCCTGGGAGTCGG
JNK	TGAAGCTCCACCACCAAAGA	CTGTGCTAAAGGAGAGGGCT
ERK1	CTACACGCAGCTGCAATACAT	TAGCAAGATCTGGATCTCTC
PIK3CA	AGTAGGCAACCGTGAAGAAAAG	GAGGTGAATTAAGATCCCTAAGA
PIK3CG	GGCGAAACGCCCATCAAAAA	GACTCCCGTGCAGTCATCC
AKT1S1	TCCTCCTCAAGAACGACGGCA	GTTCGCTCGATGACGGTCGT
AKT2	AAAGTCATCCTGGTGCG	GGGTGCCTGGTGTTCTG
JAK2	GAGCTTTGGAGTGGTTCTGTATGA	CTGGCCGTGGTAATCTTCCAT
SOCS3	CTAGAAGAGCCTATTACATCTACTC	AGGTGGCCGTTGACTGTTTT
CAMKK	ACTGGGGTCACGCTGTACTG	GGAGCCAAAGGTCAGTGAAG
PRKAG1	ATGAAGTCTCATCGCTGCTATG	ACCGTTAGTCACCAAAGCAAA
CREB2	GAGAGAAGGTGGTAGCAGCA	CTGCTCTGCCCTCTTCTTCT
PGC1A	TCGCAGTCGCAACATTTAC	TTATGAGGAGGAGTGGTGGG
NRF1	AGTGAGCCAGACTGAACACA	ATGGACCTGCTGTACTTGCT
TFAM	GCTTTGTCTACGGGTGCAAT	TCAGGGTGGATGAAAACCAT
COX1	TGGTGCCTGAGCAGGAATAGTG	ATCATCGCCAAGTAGGGTTCCG
ATP6	ACTCATTCACACCCACCACACA	CCTGCTGTAATGTTGGCTGTCA
ND4	CCTGCTGTAATGTTGGCTGTCA	GGATTATGGTTCGGCTGTGTA
ULK1	GGCAAGTTCGAGTTCTCGCG	CGACCTCCAGGTCATGTTTCT
BECN1	TGGCGGAAAATCTCGAGAAGGTCCA	TGTGCCAAATTGTCCACTGTGCCAA
P62	TACACGAGACCAGTCAACCTAAC	AGAAGATGCTTGTGCCGAG
NIX	AGCAGGGACCATAGCTCTCA	CGCTTTTCTTCAAAGCATCC

Supplemental Table 4. The list of primers used in this study.
In this table, the sequence of all primers used in the study for quantitative PCR are listed in details.
